# Quantitative evaluation of the immunodeficiency of a mouse strain by tumor engraftments

**DOI:** 10.1186/s13045-015-0156-y

**Published:** 2015-05-29

**Authors:** Wei Ye, Zhiwu Jiang, Guan-Xiong Li, Yiren Xiao, Simiao Lin, Yunxin Lai, Suna Wang, Baiheng Li, Bei Jia, Yin Li, Zhi-liang Huang, Jin Li, Fenglan Feng, Shuhua Li, Huihui Yao, Zixia Liu, Su Cao, Lin Xu, Yangqiu Li, Donghai Wu, Lingwen Zeng, Mei Zhong, Pentao Liu, Zhe-sheng Wen, Bing Xu, Yao Yao, Duanqing Pei, Peng Li

**Affiliations:** Key Laboratory of Regenerative Biology, South China Institute for Stem Cell Biology and Regenerative Medicine, Guangzhou Institutes of Biomedicine and Health, Chinese Academy of Sciences, Guangzhou, 510530 China; Guangdong Provincial Key Laboratory of Stem Cell and Regenerative Medicine, South China Institute for Stem Cell Biology and Regenerative Medicine, Guangzhou Institutes of Biomedicine and Health, Chinese Academy of Sciences, Guangzhou, 510530 China; Department of General Surgery, The Second Hospital of Yulin, Yulin, Shaanxi Province 719000 China; Department of Obstetrics and Gynecology, Nanfang Hospital, Southern Medical University, Guangzhou, 510515 China; Department of Hematology, Nanfang Hospital, Southern Medical University, Guangzhou, 510515 China; Department of Thoracic Oncology, Sun Yat-Sen University Cancer Center, Guangzhou, China; State Key Laboratory of Oncology in South China, Collaborative Innovation Center of Cancer Medicine, Guangzhou, China; State Key Laboratory of Respiratory Disease, The First Affiliate Hospital of Guangzhou Medical University, Guangzhou, China; Department of Pathology, School of Basic Medical Sciences, Guangzhou Medical University, Guangzhou, 510182 China; Department of Outpatient, The 91th Military Hospital, Jiaozuo, 454003 China; Division of Reproductive Endocrinology, The 91th Military Hospital, Jiaozuo, 454003 China; Division of General Pediatrics, The 91th Military Hospital, Jiaozuo, 454003 China; Institute of Hematology, Medical College, Jinan University, Guangzhou, 510632 China; Key Laboratory for Regenerative Medicine of Ministry of Education, Jinan University, Guangzhou, 510632 China; Wellcome Trust Sanger Institute, Hinxton, Cambridge, CB10 1HH England UK; Drug Discovery Pipeline, Guangzhou Institutes of Biomedicine and Health, Chinese Academy of Sciences, Guangzhou, 510530 China; Guangzhou Institutes of Biomedicine and Health, Chinese Academy of Sciences, 190 Kaiyuan Avenue, Science Park, Guangzhou, Guangdong 510530 China

**Keywords:** Immunodeficiency, Tumor, Leukemia, Xenograft, Allograft

## Abstract

**Background:**

The mouse is an organism that is widely used as a mammalian model for studying human physiology or disease, and the development of immunodeficient mice has provided a valuable tool for basic and applied human disease research. Following the development of large-scale mouse knockout programs and genome-editing tools, it has become increasingly efficient to generate genetically modified mouse strains with immunodeficiency. However, due to the lack of a standardized system for evaluating the immuno-capacity that prevents tumor progression in mice, an objective choice of the appropriate immunodeficient mouse strains to be used for tumor engrafting experiments is difficult.

**Methods:**

In this study, we developed a tumor engraftment index (TEI) to quantify the immunodeficiency response to hematologic malignant cells and solid tumor cells of six immunodeficient mouse strains and C57BL/6 wild-type mouse (WT).

**Results:**

Mice with a more severely impaired immune system attained a higher TEI score. We then validated that the NOD-*scid-IL2Rg−/−* (NSI) mice, which had the highest TEI score, were more suitable for xenograft and allograft experiments using multiple functional assays.

**Conclusions:**

The TEI score was effectively able to reflect the immunodeficiency of a mouse strain.

**Electronic supplementary material:**

The online version of this article (doi:10.1186/s13045-015-0156-y) contains supplementary material, which is available to authorized users.

## Background

Research on human diseases has relied on experiments using immunodeficient mouse models [1]. The derivations of nude and severe combined immunodeficiency (*scid*) mice, which are widely used for xenotransplantation, were milestones in the development of immunodeficient mice. However, although nude mice lacked T cells, they harbored B cells and natural killer (NK) cells and did not allow lasting human cell reconstitution [2]. The limitations that impeded human cell engraftment in *scid* and recombination-activating 2 deficient (*Rag2−/−*) mice included the remaining mouse T and B cells and high levels of host NK cells [3, 4]. The development of NOD.Cg-*Prkdc*^*scid*^ (NOD-*scid*) mice with lower levels of NK cells and additional innate immune defects allowed higher levels of human cell engraftment, but the mice were still not ideal [5]. A major breakthrough in the generation of humanized mice was the development of immunodeficient *IL2Rg−/−* mice, such as the NOD/*ShiLtSz-scid/IL2Rγ*^null^ (NSG) and NOD/*ShiJic-scid/IL2Rγ*^null^ (NOG) strains. These mice withstood greatly increased engraftments of human tissues (hematopoietic stem cells (HSCs) and peripheral blood mononuclear cells (PBMCs)) than all previously developed immunodeficient humanized mouse models [6, 7]. Cancer cells and the host immune system constantly interact with one another in the tumor microenvironment [8, 9]. Clinical data have demonstrated that immunodeficient individuals are susceptible to a dramatic increase in tumor incidence. For example, the occurrence of leukemia was higher in immunodeficient patients compared with the general population [10]. Consistently with immunodeficient patients, STAT-1−/− and RAG−/− mice showed a significantly increased incidence of observable cancers compared with their non-immunodeficient counterparts [11, 12]. Thus, a greater severity of immune deficiency led to a greater degree of tumor growth in immunodeficient mice.

Following the development of large-scale mouse knockout programs [13] and genome-editing tools, such as zinc-finger nuclease (ZFN) [14], transcription activator-like effector nuclease (TALEN) [15–17] and the type II clustered, regularly interspaced, short palindromic repeat (CRISPR)-associated (Cas) system [18–20], it has become increasingly efficient to generate genetically modified mouse strains that cannot easily be generated using traditional hybridization [21]. However, because no standardized system for evaluating immunodeficiency in mice currently exists, an objective comparison of the immunodeficiency of various immunodeficient mouse strains is difficult.

In this study, we developed a tumor engraftment index (TEI), using six representative immunodeficient mouse models, combining the hematopoietic and solid tumor model and the allograft and xenograft models, and created a statistical formula for a simple and accurate method to quantify the immunodeficiency of mouse strains. We showed that the NOD-*scid-IL2Rg−/−* (NSI) mice had the highest TEI scores in both the xenograft and allograft tests. Moreover, we validated the TEI scoring results using human-derived HSC, human bone marrow/liver/thymus (BLT), single primary B cell acute lymphoblastic leukemia (B-ALL) cell transplantation models, and primary tumors from lung cancer patients.

## Results

### Assessing the ability to prevent the engraftment of hematological tumors

Immunodeficiency is positively correlated with the capacity for tumor engraftment [22, 23]. We chose C57BL/6 wild-type (WT) and six well-characterized immunodeficient mouse strains, namely nude [2], *scid* [3], NOD-*scid* [5], B6.129S4-*IL2Rg*−/− (*IL2Rg−/−*) [24], *Rag2−/−* [4], and NOD-*scid-IL2Rg−/−* [5, 7] mice, to assess immunodeficiency. Because the NSG and NOG strains are not commercially available in China, we generated the NOD-*scid-IL2Rg−/−* (NSI) strain, which did not harbor T, B, or NK cells, by TALEN-mediated gene targeting in the NOD background [25].

**Fig. 1:**
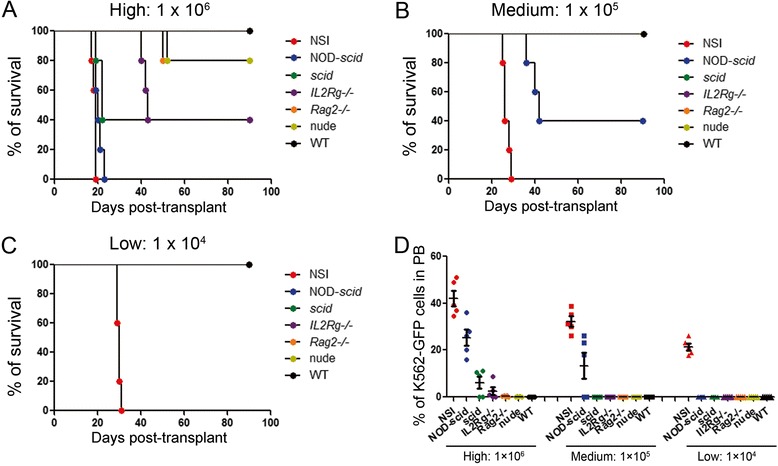
Assessing the ability to withstand a leukemic xenograft in immunodeficient mice. Kaplan-Meyer survival analysis of NSI, *IL2Rg−/−*, NOD-*scid*, *scid*, *Rag2−/−*, nude, and WT mice injected with a high number (1 × 10^6^, **a**), medium number (1 × 10^5^, **b**), and low number (1 × 10^4^, **c**) of K562-GFP cells. **d** The percentages of K562-GFP cells in the peripheral blood (*PB*) of each mouse in the same experiments in (**a**). *Bars* represent the mean percentages of human K562-GFP cells in the PB of mice from each strain (*n* = 5)

For an accurate quantification, we first evaluated the immunodeficiency of a mouse strain by measuring its ability to prevent hematologic tumor engraftment. We injected K562-GFP cells [26], a human chronic myeloid leukemia cell line with Philadelphia chromosome, which constitutively expressed green fluorescent protein (GFP) (Additional file 1: Figure S1) into the seven mouse strains without any preconditioning, and assessed the percentages of tumor cells in certain tissues of the recipients. Three groups of mice (five mice per group) were assayed with a high number (1 × 10^6^, H), medium number (1 × 10^5^, M), and low number (1 × 10^4^, L) of grafts, respectively (Additional file 2: Figure S2). The survival curves of each group of the seven mouse strains are shown in Fig. 1. The median survival time was 19 days in NSI mice, 21 days in NOD-*scid* mice, 23 days in *scid* mice, and 45 days in *IL2Rg−/−* mice when 1 × 10^6^ K562-GFP cells were injected (Fig. 1a). No K562 cells were detected in WT mice after transplantation (Fig. 1a). After the injection of 1 × 10^5^ K562-GFP cells, successful reconstitution of leukemia was observed in NSI (medium survival time 27 days) and NOD-*scid* (medium survival time 48 days) mice (Fig. 1b), but not in *IL2Rg−/−*, *scid*, *Rag2−/−*, nude, or WT mice. The injection of 1 × 10^4^ K562-GFP cells reconstituted leukemia in the NSI mice, whereas leukemia cells were under detectable in NOD-*scid*, *IL2Rg−/−*, *scid*, *Rag2−/−*, nude, or WT mice (Fig. 1c). Because K562 cells were not detected in the bone marrow (BM) or spleen (SP) of the seven strains tested, we then measured the percentages of K562 cells in the peripheral blood (PB) of each mouse and found that the engraftment efficiencies were highest in the NSI mice, followed by the NOD-*scid*, *IL2Rg−/−*, *scid*, *Rag2−/−*, and nude mice, in that order (Fig. 1d).

**Fig. 2:**
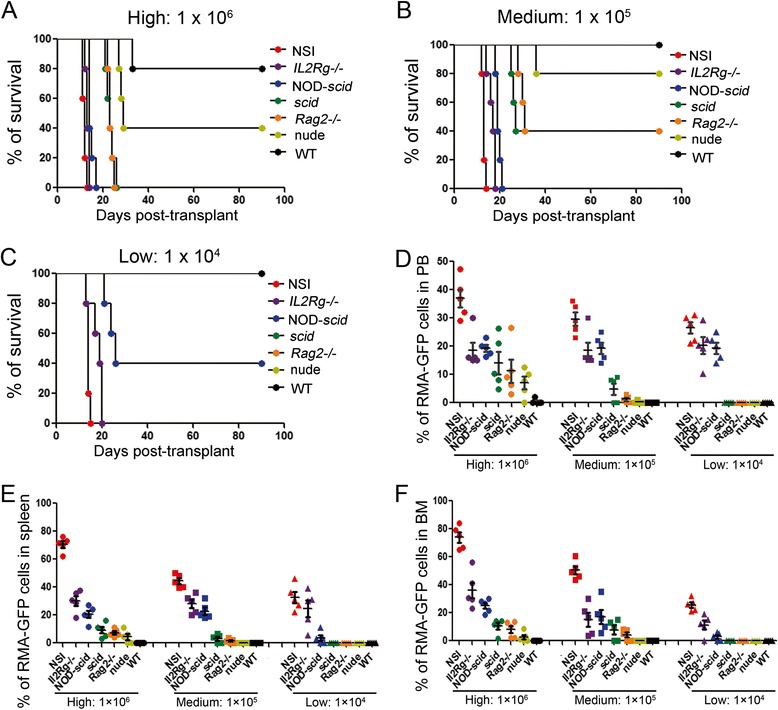
Assessing the ability to withstand a leukemic allograft in immunodeficient mice. Kaplan-Meyer survival analysis of NSI, *IL2Rg−/−*, NOD-*scid*, *scid*, *Rag2−/−*, nude, and WT mice injected with a high number (1 × 10^6^, **a**), medium number (1 × 10^5^, **b**), and low number (1 × 10^4,^ **c**) of RMA-GFP cells. **c** Level of RMA-GFP cells in peripheral blood (*PB*, **d**), spleen (*SP*, **e**), and bone marrow (*BM*, **f**) of each mouse with reference to (**a**). *Bars* represent the mean percentages of human K562-GFP cells in the PB, SP, and BM of mice from each strain (*n* = 5)

Similarly, we measured the immunodeficiency of the seven strains for hematologic allografts by injecting RMA-GFP cells [27], which continuously expressed GFP (Additional file 1: Figure S1 and Additional file 2: Figure S2). The lifespan of each strain after tumor transplantation increased in the sequential order of NSI, *IL2Rg−/−*, NOD-*scid*, *scid*, *Rag2−/−*, nude, and WT after 1 × 10^6^ RMA-GFP cells were injected (Fig. 2a). The injection of 1 × 10^5^ RMA-GFP cells did not reconstitute tumors in WT mice (Fig. 2b). After the injection of 1 × 10^4^ RMA-GFP cells, successful engraftments were detected in only NSI, *IL2Rg−/−*, and NOD-*scid* mice (Fig. 2c). We measured the percentages of RMA-GFP in the PB (Fig. 2d), SP (Fig. 2e), and BM (Fig. 2f) of each mouse.

### Assessing the ability to prevent the engraftment of solid tumor

To evaluate the feasibility of the seven mouse strains for solid tumor xenografts, we measured the immunodeficiency of a mouse strain by testing its ability to prevent solid tumor engraftment. We subcutaneously injected the six immunodeficient mouse strains and WT mice with A549 cells (for xenografts), a human adenocarcinoma alveolar basal epithelial cell line [28], or B16F10 cells (for allografts), which are a murine skin melanoma [29], to assess the tumor mass. Three groups of mice (five mice per group) were assayed with a high number (1 × 10^6^, H), medium number (1 × 10^5^, M), and low number (1 × 10^4^, L) of grafts, respectively (Additional file 2: Figure S2). Twenty days (for allografts) or 30 days (for xenografts) after transplantation, the engraftment efficiencies of the tumor cells were determined by measuring the mass of the tumor under the skin.

**Fig. 3:**
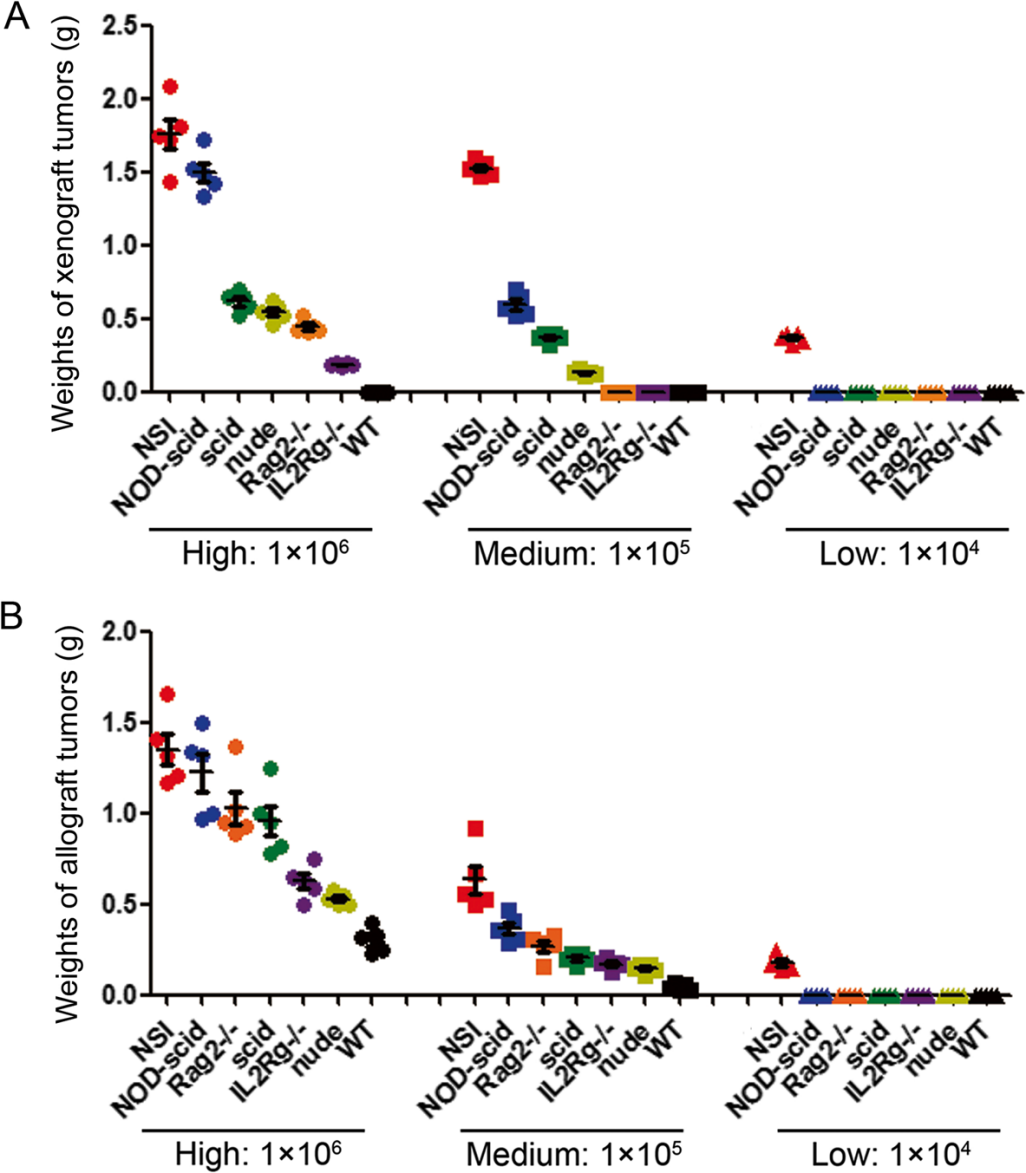
Assessing the ability to withstand a solid tumor in immunodeficient mice. The weight of the solid xenografts (**a**) and allografts (**b**) in NSI, *IL2Rg−/−*, NOD-*scid*, *scid*, *Rag2−/−*, nude, and WT mice transplanted with a high number (1 × 10^6^), medium number (1 × 10^5^), and low number (1 × 10^4^) of A549 or B16F10. *Bars* represent the mean weight of grafts from mice of each strain (*n* = 5)

Notably, we found that 1 × 10^4^ A549 cells were able to form tumors in the NSI mice, whereas 1 × 10^5^ A549 cells were required for successful engraftments in the NOD-*scid*, *scid*, and nude mice (Fig. 3a). However, 1 × 10^6^ A549 cells were needed to induce tumorigenesis in *Rag2−/−* and *IL2Rg−/−* mice. No tumors were observed in WT mice (Fig. 3a). Similarly, we measured the immunodeficiency of the seven strains for allografts by injecting B16F10 cells into five mice of each strain (Additional file 2: Figure S2). Consistent with the A549 xenograft results, tumors were detected in NSI mice after the injection of as few as 1 × 10^4^ B16F10 cells (Fig. 3b). The weights of the tumors increased in the sequential order of WT, nude, *IL2Rg−/−*, *scid*, *Rag2−/−*, NOD-*scid*, and NSI mice after the injection of 1 × 10^5^ or 1 × 10^6^ B16F10 cells (Fig. 3b).

### Formula to quantify the immunodeficiency of the immunodeficient mice

The capacities of both hematologic and solid tumor induction in the seven mouse strains evaluated suggested that the immunodeficiency was positively correlated with the capacity for tumor engraftment, which was also supported by previous studies [22, 23]. To quantify the immunodeficiency of a specific mouse strain, we developed a tumor engraftment index (TEI) that is negatively correlated to its capacity to prevent the development of non-self tumor cells. For a given time of death, TEI is proportional to the engraftment efficiency. However, for a given engraftment efficiency, TEI is inversely proportional to the time of death of the mice injected with tumor. For example, the TEI of WT or normal mice having a healthy immune system was zero; the TEI of mice with a severely impaired immune system was substantially higher. Therefore, we propose that the TEI of an individual mouse can be calculated by dividing its tumor engraftment by its lifespan after injection with the tumor cells. The quantification of tumor engraftments varies between hematologic tumors and solid tumors.

For the hematologic tumor engraftment index, ${I}_{\mathrm{strain}-\mathrm{hematologic}\;\mathrm{tumor}-n}=\frac{\mathrm{Gi}}{\mathrm{Di}}$ where “strain” is the name of the immunodeficient mice; “hematologic tumor” is the name of the tumor cells; *n* denotes the number of individuals; “Gi” is the sum of the percentages of tumor cells in the peripheral blood (*G*_PB_), bone marrow (*G*_BM_), and spleen (*G*_SP_) of an individual mouse when the mouse is morbid or killed; and “Di” is the lifespan of the individual mouse after injection of the tumor cells.

For the solid tumor engraftment index, ${I}_{\mathrm{strain}-\mathrm{solid}\;\mathrm{tumor}-n}=\frac{\mathrm{Wi}}{D}$ where “strain” is the name of the immunodeficient mice, “solid tumor” is the type of tumor cells for transplantation, *n* denotes the number of individuals, “Wi” is the weight of the graft in the mouse when the mouse is morbid or killed, and *D* is the survival time of the mouse after injection of the tumor.

The final score of the TEI is the average of *In* of hematologic and solid tumors:

$$ {I}_{\mathrm{strain}-\mathrm{allograft}-n}=\left({I}_{\mathrm{strain}-\mathrm{R}\mathrm{M}\mathrm{A}-n}+{I}_{\mathrm{strain}-\mathrm{B}16\mathrm{F}10-n}\right)/2 $$

$$ {I}_{\mathrm{strain}-\mathrm{xenograft}-\mathrm{n}}=\left({I}_{\mathrm{strain}-\mathrm{k}562-\mathrm{n}}+{I}_{\mathrm{strain}-\mathrm{A}549-n}\right)/2 $$

**Fig. 4:**
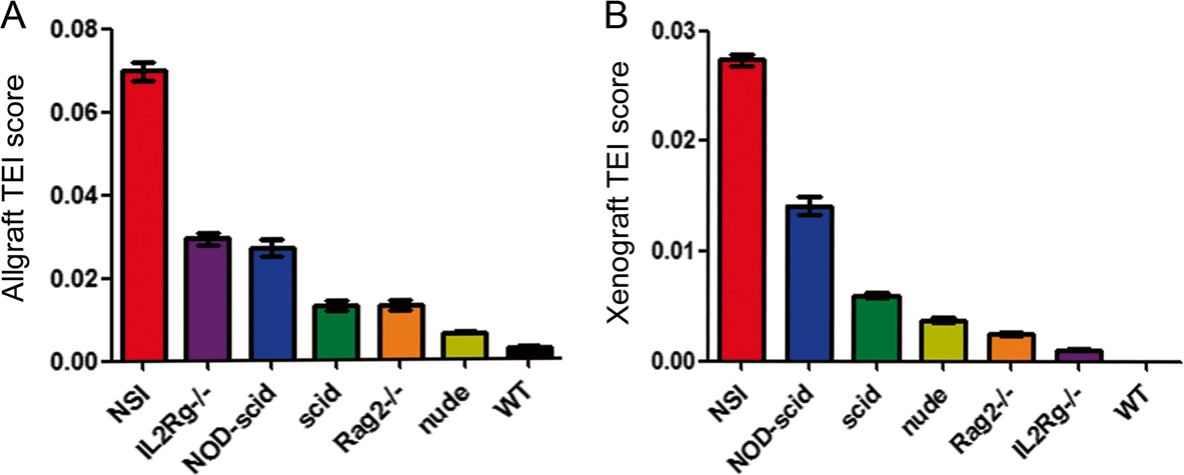
Final TEI score. Summary of the final TEI score of NSI, *IL2Rg−/−*, NOD-*scid*, *scid*, *Rag2−/−*, nude, and WT mice in the allograft (**a**) and xenograft (**b**) assay

To improve the convenience of calculation of TEI scores, we designed the website (http://www.nsitei.com) based on the TEI equation in Table 1. According to the TEI equation, we calculated the TEI scores for each strain (Additional file 3: Table S1 and Additional file 4: Table S2) and found that NSI mice had the highest TEI scores in both the xenograft and allograft tests, followed by *scid*, nude, and WT mice. Interestingly, the allograft TEI scores of *IL2Rg−/−* were higher than those of NOD-*scid* and *scid* mice (Fig. 4a), whereas *IL2Rg−/−* had lower TEI scores than NOD-*scid* and *scid* mice in the xenograft tests (Fig. 4b). The recent observations of greater leukemogenic engraftment in NSG mice than in NOD-*scid* mice [22], and of the greater susceptibility to tumor formation of NOG mice than *nude* and *scid* mice [23], indicated that the TEI scoring system provides an efficient method to assess the tumor engraftment efficiency of immunodeficient mice.

**Table 1:** Equation for calculating TEI scores

TEI score for RMA^a^																	
Mouse number	H				M				L				TEI_H_	TEI_M_	TEI_L_	TEI_RMA_	Average of TEI_RMA_
	*G*_PB_	*G*_SP_	*G*_BM_	*D*	*G*_PB_	*G*_SP_	*G*_BM_	*D*	*G*_PB_	*G*_SP_	*G*_BM_	*D*	(*G*_PB_ + *G*_SP_ + *G*_BM_)/*D*	(*G*_PB_ + *G*_SP_ + *G*_BM_)/*D*	(*G*_iPB_ + *G*_SP_ + *G*_BM_)/*D*	(TEI_H_ + TEI_M_ + TEI_L_)/3	
1	0	0	0	1	0	0	0	1	0	0	0	1	0	0	0	0	0
2	0	0	0	1	0	0	0	1	0	0	0	1	0	0	0	0	
3	0	0	0	1	0	0	0	1	0	0	0	1	0	0	0	0	
4	0	0	0	1	0	0	0	1	0	0	0	1	0	0	0	0	
5	0	0	0	1	0	0	0	1	0	0	0	1	0	0	0	0	
TEI score for B16F10^b^																	
Mouse number	H		M		L		TEI_H_	TEI_M_	TEI_L_	TEI_B16F10_	Average of TEI_B16F10_						
	*W*	*D*	*W*	*D*	*W*	*D*	*G*/*D*	*G*/*D*	*G*/*D*	(TEI_H_ + TEI_M_ + TEI_L_)/3							
1	0	1	0	1	0	1	0	0	0	0	0						
2	0	1	0	1	0	1	0	0	0	0							
3	0	1	0	1	0	1	0	0	0	0							
4	0	1	0	1	0	1	0	0	0	0							
5	0	1	0	1	0	1	0	0	0	0							
TEI_allograft_	0																
TEI score for K562^c^																	
Mouse number	H				M				L				TEI_H_	TEI_M_	TEI_L_	TEI_K562_	Average of TEI_K562_
	*G*_PB_	*G*_SP_	*G*_BM_	*D*	*G*_PB_	*G*_SP_	*G*_BM_	*D*	*G*_PB_	*G*_SP_	*G*_BM_	*D*	(*G*_PB_ + *G*_SP_ + *G*_BM_)/*D*	(*G*_PB_ + *G*_SP_ + *G*_BM_)/*D*	(*G*_iPB_ + *G*_SP_ + *G*_BM_)/*D*	((TEI_H_ + TEI_M_ + TEI_L_)/3)	
1	0	0	0	1	0	0	0	1	0	0	0	1	0	0	0	0	0
2	0	0	0	1	0	0	0	1	0	0	0	1	0	0	0	0	
3	0	0	0	1	0	0	0	1	0	0	0	1	0	0	0	0	
4	0	0	0	1	0	0	0	1	0	0	0	1	0	0	0	0	
5	0	0	0	1	0	0	0	1	0	0	0	1	0	0	0	0	
TEI score for A549^d^																	
Mouse number	H		M		L		TEI_H_	TEI_M_	TEI_L_	TEI_A549_	Average of TEI_A549_						
	*W*	*D*	*W*	*D*	*W*	*D*	*G*/*D*	*G*/*D*	*G*/*D*	(TEI_H_ + TEI_M_ + TEI_L_)/3							
1	0	1	0	1	0	1	0	0	0	0	0						
2	0	1	0	1	0	1	0	0	0	0							
3	0	1	0	1	0	1	0	0	0	0							
4	0	1	0	1	0	1	0	0	0	0							
5	0	1	0	1	0	1	0	0	0	0							
TEI_xenograft_	0																

^a^Fill the RMA-GFP engraftment efficiency in PB, SP, BM (*G*_PB_, *G*_SP_, *G*_BM_), and survival day (*D*) transplanted with H, M, and L of cells in table “TEI score for RMA”

^b^Fill the tumor weight of B16F10 (W) and sacrificed day (*D* = 20) transplanted with H, M, and L of cells in table “TEI score for B16F10”

^c^Fill the K562-GFP engraftment efficiency in PB, SP, and BM (*G*_PB_, *G*_SP_, *G*_BM_), and survival day (*D*) transplanted with H, M, and L cells in table “TEI score for K562”

^d^Fill the tumor weight of A549 (W) and sacrificed day (*D* = 30) transplanted with H, M, and L of cells in table “TEI score for A549”

### Hematopoietic functional assay in NSI mice

**Fig. 5:**
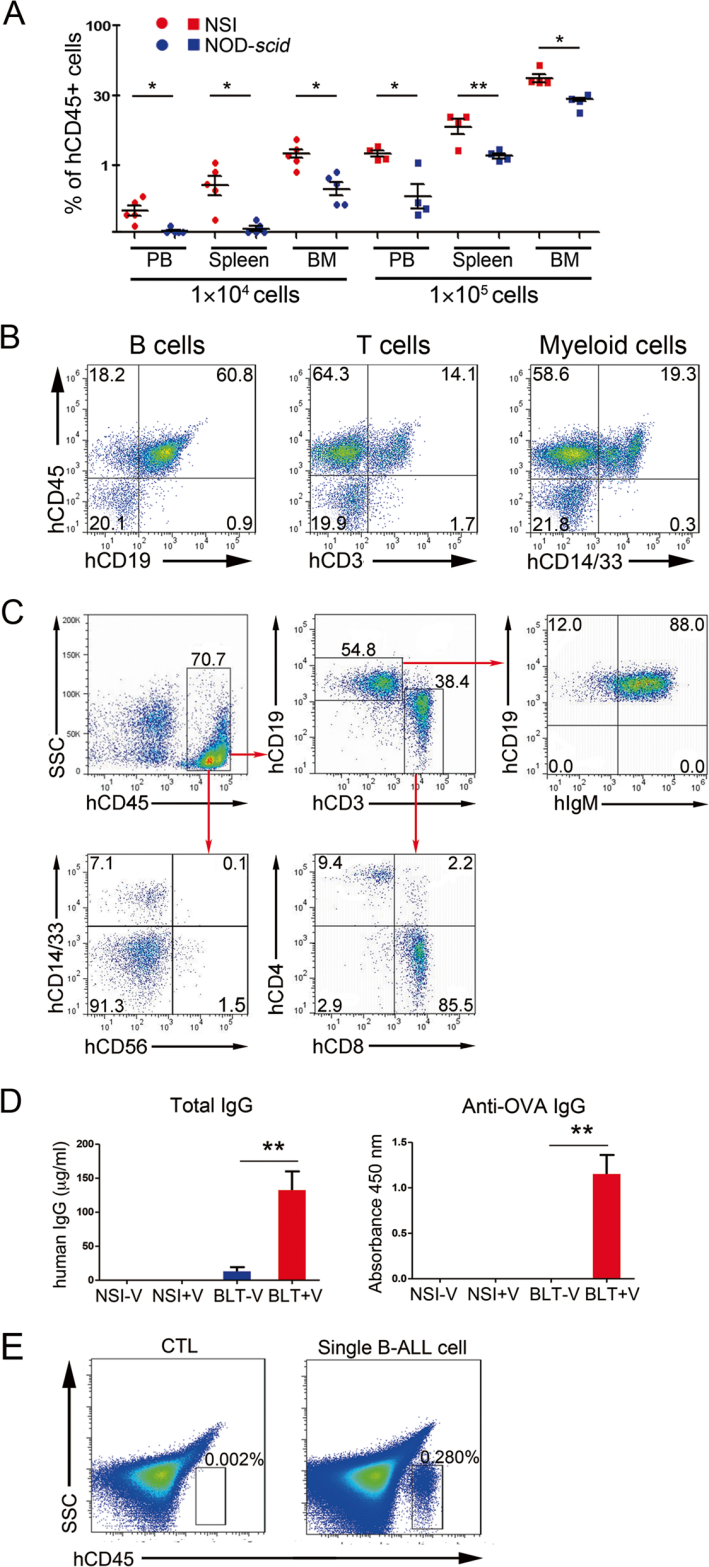
Hematopoietic functional assays of NSI mice. **a** Summary of percentages of human CD45+ cells in the PB, BM, and SP of NSI (*red plot*) and NOD-*scid* (*blue plot*) mice 20 weeks after injection with 1 × 10^4^ or 1 × 10^5^ purified human CD34+. *Bars* represent the mean percentages of human CD45+ cells in the PB, BM, and SP of mice from each group (*n* = 4 or 5 per group). *P ≤ 0.05 for bar 1 versus bar 2, bar 3 versus bar 4, bar 5 versus bar 6, bar 7 versus bar 8, and bar 11 versus bar 12; **P ≤ 0.01 for bar 9 versus bar 10. **b** Representative FACS analysis of percentages of multiple hematopoietic lineages in NSI as described in (**a**). **c** Representative fluorescence-activated cell sorting (FACS) analysis of percentages of multiple hematopoietic lineages in NSI transplanted with human cord blood CD34+/liver/thymus. **d** The level of human IgG (*left*) and OVA-specific IgG (*right*) in serum of NSI mice transplanted with human cord blood CD34+/liver/thymus. *Open bars* represent NSI mice that received human cord blood CD34+/liver/thymus (*n* = 3). Data are represented as the mean ± standard error of the mean. **P ≤ 0.01 for bar 3 versus bar 4 and bar 7 versus bar 8. **e** Representative FACS analysis of percentages of human CD45+ cells in NSI (*right*) and Nod-*scid* (*left*) transplanted with a single primary B-ALL cell

We then used three types of functional assay to validate whether NSI mice were more suitable for xenograft than NOD-*scid* mice, as suggested by the TEI results. First, we compared the human hematopoietic engraftment capacities of the NSI mice and NOD-*scid* mice by engrafting the sub-lethally irradiated mice with 1 × 10^4^ and 1 × 10^5^ of human umbilical cord blood CD34+ cells, respectively. Twelve weeks after the transplantations, the percentages of human CD45+ cells in the PB, SP, and BM of the NSI mice were significantly higher than those of the NOD-*scid* mice (Fig. 5a). Furthermore, the injected human CD34+ cells differentiated into multiple hematopoietic lineages, including B cells, T cells, and myeloid cells (Fig. 5b).

Second, we examined whether human hematopoietic progenitors were able to reconstitute a functional immune system in NSI mice. We established a humanized BLT mouse model by surgically implanting human thymus and liver fragments into kidney capsules and injecting donor-matched human hematopoietic stem/progenitor cells (CD34^+^) intravenously into NSI mice (Additional file 5: Figure S3). Twelve weeks after transplantation, we analyzed the immune system in the recipient BLT-NSI mice. We found human mature CD4+ and CD8+ T cells, CD19+ immunoglobulin (Ig)M+ mature B cells, CD56+ NK cells, and CD14/33+ monocytes/macrophages in the BLT-NSI mice (Fig. 5c). More importantly, we detected not only human IgG in the sera of the BLT-NSI mice but also ovalbumin (OVA)-specific human IgG from the sera of the mice after they were immunized with OVA peptides (Fig. 5d). Conversely, we failed to reconstitute functional human immune systems in the NOD-*scid* mice using the same method (data not shown).

Finally, we tested whether a single human primary leukemic cell was capable of reconstituting leukemia in NSI mice. Twenty weeks after the transplantation of a single primary B-ALL cell, human CD45^+^ B-ALL was reconstituted in the NSI mice, but not in the NOD-*scid* mice (Fig. 5e).

### Establishment of solid tumor patient-derived xenografts with NSI mice

**Fig. 6:**
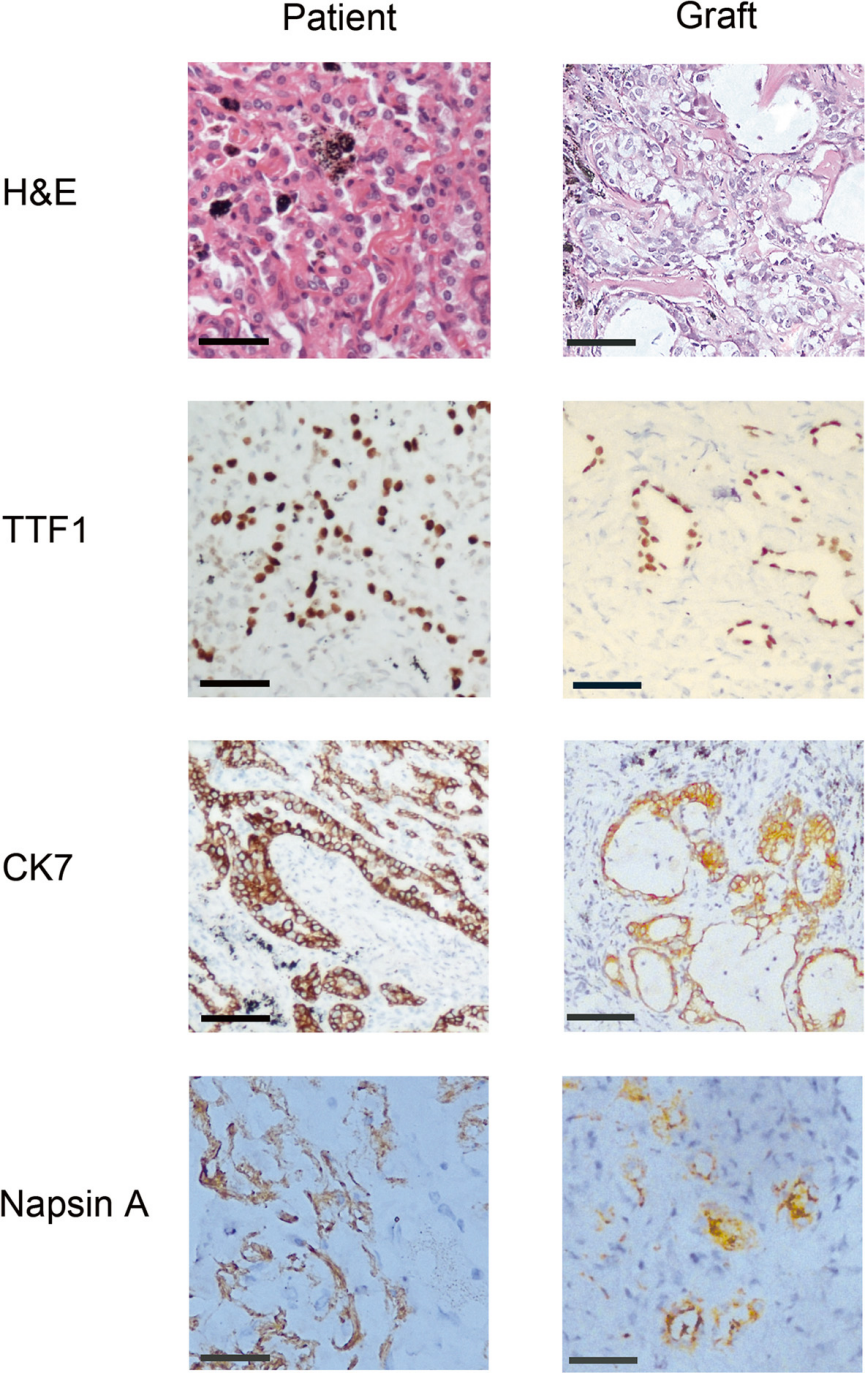
Generation and characterization of the lung cancer xenograft model with NSI mice. Representative hematoxylin and eosin- and immunohistochemistry-stained tissues of an adenocarcinoma (left) and corresponding early-generation xenografts (*right*); scale bar = 50 μm

We further examined whether NSI mice were suitable for modeling solid tumor patient-derived xenograft (PDX). Ten primary non-small cell lung cancer (NSCLC) samples from different patients were harvested and implanted subcutaneously into NSI mice (Table 2). Six of the 10 samples were successfully engrafted in NSI mice (Table 2). Therefore, the successful rate of modeling NSCLC in PDX using NSI mice was 60 %, which was higher than the 24.5 % in NOD-*scid* mice [30, 31] and 35 % in *scid* mice [32]. Interestingly, we detected murine Ly6g + CD11b + macrophages in the tumors from the NSCLC PDX models (Additional file 6: Figure S4). The tumors in the NSCLC PDX models exhibited similar morphology to that of the patient tissues from which the primary models were derived (Fig. 6). In addition, dissociated tumor cells from primary lung cancer samples were also able to reconstitute tumors with similar morphology to that of the patient tumors (Additional file 7: Figure S5).

**Table 2:** Information of patients and corresponding patient-derived xenograft mouse models

Number	Gender	Age	EGFR mutation	Pathology	Stage	TNM stage	Engrafted
1	M	60	WT	AC	IB	T2N0M0	Yes
2	M	69	WT	AC	IIA	T2N1M0	Yes
3	M	65	WT	AC	IA	T2N0M1	Yes
4	M	67	WT	LCC	IA	T2N1M0	Yes
5	F	69	G719X/exon 19 del	AC	IIB	T2N1M0	No
6	M	62	WT	AC	IV	T2N0M0	No
7	M	71	WT	SCC	IB	T2N0M0	Yes
8	M	62	WT	AC	IIIA	T2N2M0	No
9	F	40	Exon 19 del	AC	IV	T2N1M0	Yes
10	M	67	L858R	SCC	IB	T2N0M0	No

*M* male, *F* female, *AC* adenocarcinoma, *LCC* large cell carcinoma, *SCC* squamous cell carcinoma

## Discussion

Several strains of immunodeficient mice are available for basic and translational research, including BALB/c nude (nude), *scid*, NOD*-scid*, *IL2Rg−/−*, *Rag2−/−*, and NOD-*scid-IL2Rg−/−* mice. However, the choice of an appropriate immunodeficient mouse strain is difficult, because a standardized system to evaluate the immunodeficiency of mice is lacking. In this study, we proposed an index, the TEI, to represent the capacity of a mouse strain to enhance or prevent the expansion of foreign cells in vivo. We then developed a TEI scoring method to quantitatively assess the growth of tumor cell lines in different immunodeficient mouse strains. Multiple functional assays were used to validate the correlation between the immunodeficiency of a mouse strain and its TEI score. The final TEI scores of tested immunodeficient strains are helpful to select appropriate immunodeficient strains for different experiments. For example, NSI and *IL2Rg−/−* strains are more suitable for allograft studies, while NSI and NOD*-scid* strains are preferred for establishing xenografts. Thus, the TEI scoring system was shown to be a practical and convenient method to quantify the immunodeficiency of a mouse strain. It is interesting to notice that *IL2Rg−/−* mice exhibited the seemingly paradoxical TEI scores in allograft and xenograft tests. Due to different genetic mutations in NSI, NOD*-scid*, and *IL2Rg−/−* strains, development and activities of adaptive and innate immunities, including B cells, T cells, NK cells, and macrophages, are different among these strains. In addition, NK cells and T cells use different machinery to recognize tumors in both allografts and xenografts. Therefore, the different compartments of immune systems in these strains and the genetic background of tumor cells used in allografts and xenografts may explain the paradoxical TEI scores of *IL2Rg−/−* strain in allograft and xenograft tests.

The immune system not only prevents tumor growth but also attacks other non-self entities, such as viruses, fungi, and bacteria [33]. However, immune system responds to various bacterial, viral, fungal in association with toll-like receptors and much unknown innate receptors [33, 34] Thus, the TEI scoring method that only uses cancer cells for an evaluation cannot assess the immunodeficiency of a mouse strain comprehensively. In addition, it is not practical for an ordinary laboratory to use viruses or bacteria to measure the immunodeficiency of certain mouse strains, because most of the immunodeficient strains are maintained in specific pathogen-free grade animal facilities. Therefore, the current TEI scoring method is more suitable for oncologists than microbiologists. To evaluate immunodeficiency more comprehensively and accurately, more parameters need to be incorporated into the TEI scoring system, including the ability to defend against bacterial or viral infections.

## Conclusions

Due to its simplicity and accuracy, the TEI can be used widely to help researchers to decide which strain is most suitable for their xenograft experiments. In addition, the TEI scoring system may be used to evaluate at least partially the immunodeficiency of genetically modified mice and other species in oncologic studies in the future.

## Material and methods

### Mice

Animal experiments were performed in the Laboratory Animal Center of the Guangzhou Institutes of Biomedicine and Health (GIBH), and all animal procedures were approved by the Animal Welfare and Ethical Committee of GIBH (the ethical process number: N2014050). *IL2Rg−/−* mice were purchased from Jackson Laboratories. NOD-*scid*, *scid*, nude, and WT mice were purchased from Vital River Laboratory Animal Technology Co. (Beijing). *Rag2−/−* mice were purchased from HFK Bioscience Co. (Beijing). We generated NOD-*scid*-*IL2Rg−/−* by TALEN-mediated gene targeting in the NOD background and named the new strain of immunodeficient mice NSI. The absence of T, B, and NK cells in the PB, SP, and BM of NSI mice was determined by Accuri™ C6 (BD Biosciences, San Jose, CA, USA). All of the mice were bred and maintained in specific pathogen-free grade cages and provided with autoclaved food and water.

### Cell culture

RMA cells were obtained from Professor Pengtao Liu (Sanger Institute, Cambridge, UK), and K562 cells were obtained from the American Type Culture Collection (ATCC, Manassas, VA, USA). RMA, A549, and B16F10 cells were maintained in RPMI-1640 medium (Gibco, New York, NY, USA) with 10 % fetal bovine serum (FBS; Biochrom, Australia). K562 cells were maintained in Iscove’s Modified Dulbecco Medium (Thermo Scientific, MA, USA) with 10 % FBS. The 293T cells used for lentivirus packaging were kindly provided by Professor Duanqing Pei (GIBH) and maintained in Dulbecco’s Modified Eagle’s medium (Gibco, New York, NY, USA) with 10 % FBS. All primary samples were obtained with informed consent for research purposes, and the procedures were approved by the Research Ethics Board of the GIBH. All cells were cultured at 37 °C in 5 % carbon dioxide and a normal level of oxygen.

### Lentivirus production and transduction

Twenty-four hours before the transfection, the HEK-293T cells in logarithmic growth phase were trypsinized, and the cell density was adjusted to 1.0 × 10^6^ cells/mL with complete culture medium. The cells were reseeded into 15-cm cell culture dishes and cultured for 24 h before transfection. When the cells were 90–95 % confluent on the day of transfection, the recombinant viral vector encoding GFP and the two packaging plasmids psPAX2 and pMD2.G were co-transfected into the HEK-293T cells using Lipofectamine 2000 (Invitrogen, New York, NY, USA) according to the manufacturer’s instructions. At 48 h after transfection, the culture medium was collected and centrifuged at 4000 × *g* at 4 °C for 10 min to remove any cellular debris. The supernatant was filtered through a 0.45-μm filter and collected to transfect the RMA and K562 cells. The RMA-GFP+ and K562-GFP+ cells were sorted out using FACSAria™ II (Becton Dickinson, San Jose, CA, USA) for culture.

### Flow cytometry analysis

Cells were isolated from the PB, BM, and SP for flow cytometric analyses. To analyze the human and mouse cells, the cells were labeled with anti-hCD3-fluorescein isothiocyanate, anti-hCD4-allophycocyanin (APC), anti-hCD8a-peroxidase (PE), anti-hCD14-PE, anti-hCD33-PE, anti-hCD71-PE, anti-hCD56-APC, anti-hIgM-eFluor 450, anti-hCD45-Percp Cy 5.5, anti-mCD4-PE, anti-mCD8-peridinin chlorophyll (Percp) Cy 5.5, anti-mCD19-APC, anti-mB220-Percp, anti-mCD3-APC, anti-mNKp46-PE, anti-mCD71-PE, anti-m TER119-APC, anti-mCD11b-PE, and anti-mGr-1-APC. All of the antibodies were obtained from eBioscience (San Diego, CA, USA) unless specifically stated. Flow cytometric analysis was performed using Accuri C6 or FACSAria™ II. All of the data were analyzed with FlowJo software (Tree Star, Inc., Ashland, OR, USA).

### Engraftments of leukemia and solid tumors

For a direct comparison of susceptibility to cancer cell engraftment, 1 × 10^4^ (L), 1 × 10^5^ (M), or 1 × 10^6^ (H) of RMA-GFP+, K562-GFP+, A549, or B16F10 cells suspended in 0.2 mL of phosphate buffer solution were injected into the tail vein of five NSI, *IL2Rg−/−*, NOD-*scid*, *scid*, nude, and WT mice. Similarly, 1 × 10^4^ (L), 1 × 10^5^ (M), or 1 × 10^6^ (H) of A549 or B16F10 cells suspended in 0.2 mL of phosphate buffer solution were injected subcutaneously into five NSI, *IL2Rg−/−*, NOD-*scid*, *scid*, nude, and WT mice. The end points proposed were based on animal models in widespread use [35]. The engraftment of leukemia was measured by analyzing the GFP+ cells in the PB every week or when the mice were moribund at a maximum of 90 days after grafting. Due to regulations of research animal welfare, we terminated the mice once the diameters of their tumor reach 1.2 cm. 1B16F10 and A549 cells took 20 and 30 days, respectively, to develop into a 1.2-cm tumor in NSI mice.

### Reconstitution of human hematopoiesis in NSI mice

The human cord blood was collected at the South China Medical University (SCMU) Department of Gynecology and Obstetrics with informed consent for research purposes only, and this process was monitored by the Institutional Review Boards of the SCMU. Human cord whole white blood cells were isolated using Lymphoprep (Stemcell Technologies, Canada) according to the manufacturer’s instructions. Human cord blood CD34+ cells were enriched via magnetic cell sorting (Miltenyi, Bergisch Gladbach, Germany). A total of 1 × 10^4^ or 1 × 10^5^ uncultured human CD34+ cells were pooled together and injected intravenously via the retro-orbital route into sub-lethally irradiated (1.5 Gy) 8–10-week-old NSI mice. After 12 weeks, the mice were killed, and the bone marrow from femurs was analyzed by flow cytometry for the presence of human CD45+ and blood lineage cells. In our engraftment assay, the mice were considered to have been engrafted successfully when ≥0.1 % human CD45+ cells were detected in the bone marrow 8 weeks after transplant.

### BLT mice

Human fetal thymus and liver tissues were obtained from the SCMU. Human fetal thymus and liver fragments measuring about 2 mm^3^ were implanted under the renal capsule of sub-lethally irradiated NSI mice. The mice also received CD34+ cells (5 × 10^5^/mouse, intravenously) purified from the same donor on the day of human thymus/liver transplantation. After 12 weeks, PBMCs from the recipient mice were analyzed by flow cytometry for the presence of human CD45+ and blood lineage cells.

### Single-cell preparation

PBMCs were collected from B-ALL patients (SCMU Department of Gynecology). To remove the red blood cells, the cells were treated with 1 × RBC lysis buffer (eBioscience) according to the manufacturer’s instructions. To isolate a single cell from the B-ALL patients’ peripheral blood, single-cell suspensions were prepared by standard procedures. The single cell was then transplanted into NSI mice.

### Enzyme-linked immunosorbent assay

The human Ig concentration in recipient serum was measured by using a human Ig assay kit (ab100547, Abcam, Cambridge, UK). To detect OVA-specific human IgG antibodies, five recipient BLT mice were immunized twice every 2 weeks with 100 μg of OVA (Sigma, St Louis, MO, USA) that was emulsified in aluminum hydroxide (Sigma). OVA was plated at a concentration of 10 μg/mL in 96-microtiter wells at 4 °C overnight. After washing and blocking with bovine serum albumin, the serum samples were incubated in the plate for 1 h. Antibodies binding OVA were then measured by a standard enzyme-linked immunosorbent assay.

### Establishment of xenografts

Surgical tumor samples were obtained from the Sun Yat-Sen University Cancer Center (Guangzhou, China), and cut into 3–4-mm pieces and mixed then transplanted subcutaneously within 30 min into three to six immunodeficient NSI mice. The tumor-implanted mice were observed daily for 90 days. Tumors were measured once a week by caliper to determine the subcutaneous growth rate. At a size of about 1 cm^3^, tumors were removed and passed on to other NSI mice. Tumors were passed on no more than 10 times.

### Statistical analyses

Data were analyzed using GraphPad Prism 5 with the Student’s *t* test. *P* values less than 0.05 were considered statistically significant.

## Additional files

Additional file 1: Figure S1. Establishment of K562-GFP and RMA-GFP cell line. A. Flow chart of establishing K562-GFP and RMA-GFP cells that that constitutively expressed green fluorescent protein (GFP). B. Representative fluorescence-activated cell sorting plots show K562-GFP and RMA-GFP cells before and after GFP+ enrichment. Additional file 2: Figure S2. Experimental design for assessing the capabilities of leukemic (top) or solid (bottom) grafts in immunodeficient mice. Three groups of mice (five mice per group) were assayed; a high number (1 × 10^6^, H), medium number (1 × 10^5^, M), and low number (1 × 10^4^, L) of grafts (K562-GFP, RMA-GFP, A549, and B16F10) were injected into NSI, *IL2Rg−/−*, NOD-*scid*, *scid*, *Rag2−/−*, nude, and WT mice. Additional file 3: Table S1. The final TEI scores of NSI, NOD-*scid*, *scid*, nude, *Rag2−/−*, *IL2Rg−/−*, and WT mice measured by xenograft experiments. Additional file 4: Table S2. The final TEI score of NSI, NOD-*scid, IL2Rg−/−*, *Rag2−/−*, *scid*, nude and WT mice measured by allograft experiments. Additional file 5: Figure S3. Flow chart of the functional verification of NSI mice by establishing a BLT model. Sub-lethally irradiated NSI mice were transplanted with human fetal liver and thymus tissue from the same human donors, and engrafted with autologous CD34+ hematopoietic stem cells. BLT-NSI mice were tested with flow cytometry 12 weeks after engraftment. At 12 weeks, BLT-NSI mice were immunized with OVA twice and then the serum was analyzed for human IgG. Additional file 6: Figure S4. Tumors dissected from NSCLC PDX mice contained the bone marrow-derived cells of the hosts. Representative plots of FACS analysis show dissociated cells from the tumors contained both human cells (HLA+) and murine cells (MHC I+) that were further subjected for analysis of murine Ly6g and CD11b expression. Additional file 7: Figure S5. Generation and characterization of the lung cancer xenograft model using dissociated tumour cells in NSI mice. Primary tumors from NSCLC patients were digested and dissociated with trypsin and were subsequently injected into NSI mice. Representative hematoxylin and eosin-stained tissues of an adenocarcinoma (left) and corresponding early-generation xenografts (right); scale bar = 50 μm.

## Abbreviations

TEI: Tumor engraftment index
WT: C57BL/6 wild type
*Scid*: Severe combined immunodeficiency
*Rag2−/−*: Recombination-activating 2 deficient
NOD-*scid*: NOD.Cg-*Prkdc*^*scid*^
*IL2Rg*−/−: B6.129S4-*IL2Rg*−/−
NK: Natural killer
NSG: NOD/*ShiLtSz-scid/IL2Rγ*^null^
NOG: NOD/*ShiJic-scid/IL2Rγ*^null^
NSI: NOD-*scid-IL2Rg*−/−
PBMCs: Peripheral blood mononuclear cells
HSCs: Hematopoietic stem cells
ZFN: Zinc-finger nuclease
TALEN: Transcription activator-like effector nuclease
CRISPR: Clustered regularly interspaced short palindromic repeats
Cas: CRISPR associated
BLT: Bone marrow/liver/thymus
B-ALL: B cell acute lymphoblastic leukemia
PDX: Patient-derived xenograft
WT: Wild type
BM: Bone marrow
SP: Spleen
PB: Peripheral blood
OVA: Ovalbumin
NSCLC: Non-small cell lung cancer
