# Supplementary figures and images for: Quantitative evaluation of the immunodeficiency of a mouse strain by tumor engraftments
Source: J Hematol Oncol. 2015 May 29;8:59. doi: 10.1186/s13045-015-0156-y (PMC4478639; doi:10.1186/s13045-015-0156-y)

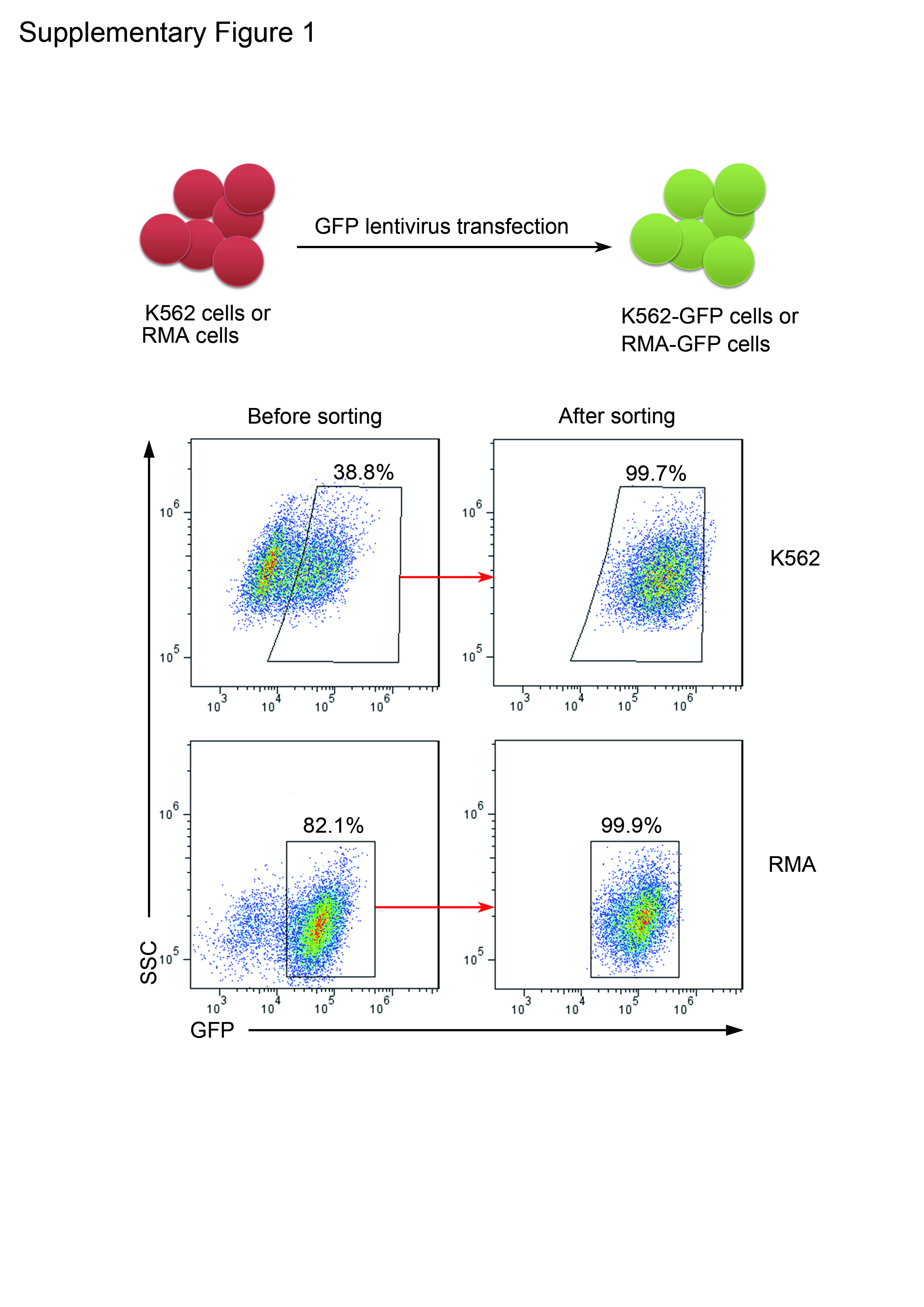

Supplement: Additional file 1: Figure S1. — Establishment of K562-GFP and RMA-GFP cell line. A. Flow chart of establishing K562-GFP and RMA-GFP cells that that constitutively expressed green fluorescent protein (GFP). B. Representative fluorescence-activated cell sorting plots show K562-GFP and RMA-GFP cells before and after GFP+ enrichment. [file 13045_2015_156_MOESM1_ESM.jpg]

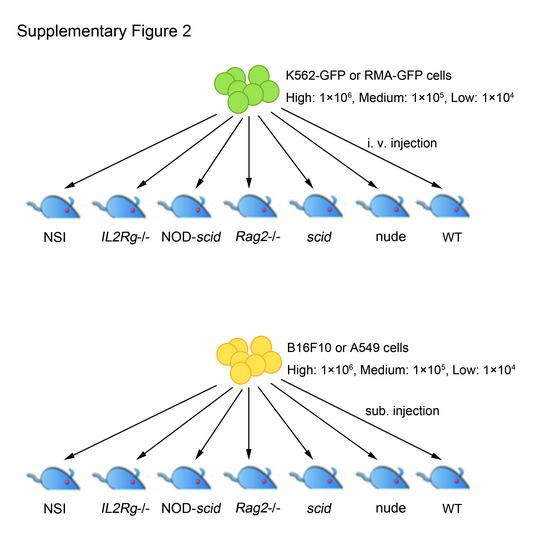

Supplement: Additional file 2: Figure S2. — Experimental design for assessing the capabilities of leukemic (top) or solid (bottom) grafts in immunodeficient mice. Three groups of mice (five mice per group) were assayed; a high number (1 × 10^6^, H), medium number (1 × 10^5^, M), and low number (1 × 10^4^, L) of grafts (K562-GFP, RMA-GFP, A549, and B16F10) were injected into NSI, IL2Rg−/−, NOD-scid, scid, Rag2−/−, nude, and WT mice. [file 13045_2015_156_MOESM2_ESM.png]

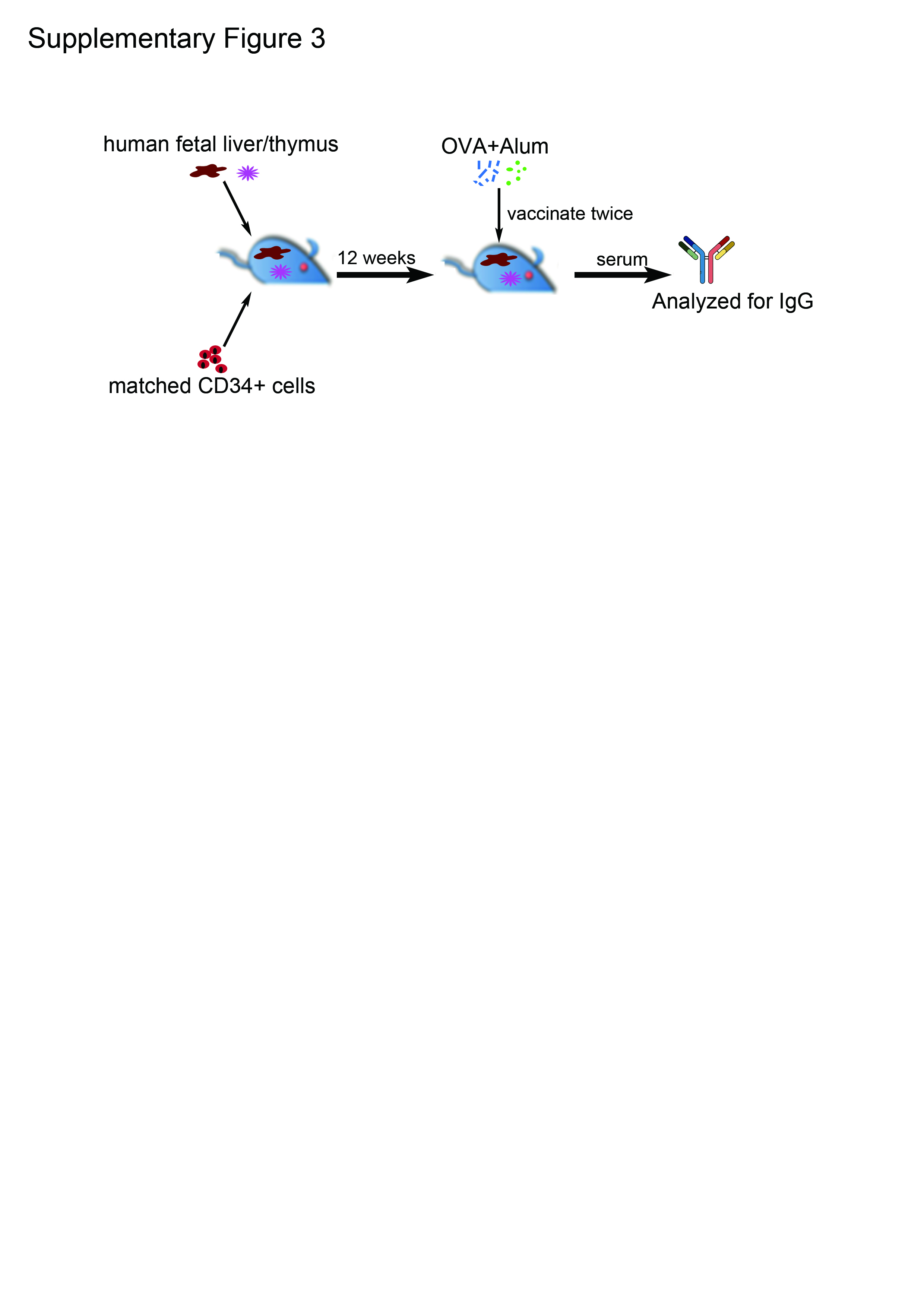

Supplement: Additional file 5: Figure S3. — Flow chart of the functional verification of NSI mice by establishing a BLT model. Sub-lethally irradiated NSI mice were transplanted with human fetal liver and thymus tissue from the same human donors, and engrafted with autologous CD34+ hematopoietic stem cells. BLT-NSI mice were tested with flow cytometry 12 weeks after engraftment. At 12 weeks, BLT-NSI mice were immunized with OVA twice and then the serum was analyzed for human IgG. [file 13045_2015_156_MOESM5_ESM.jpg]

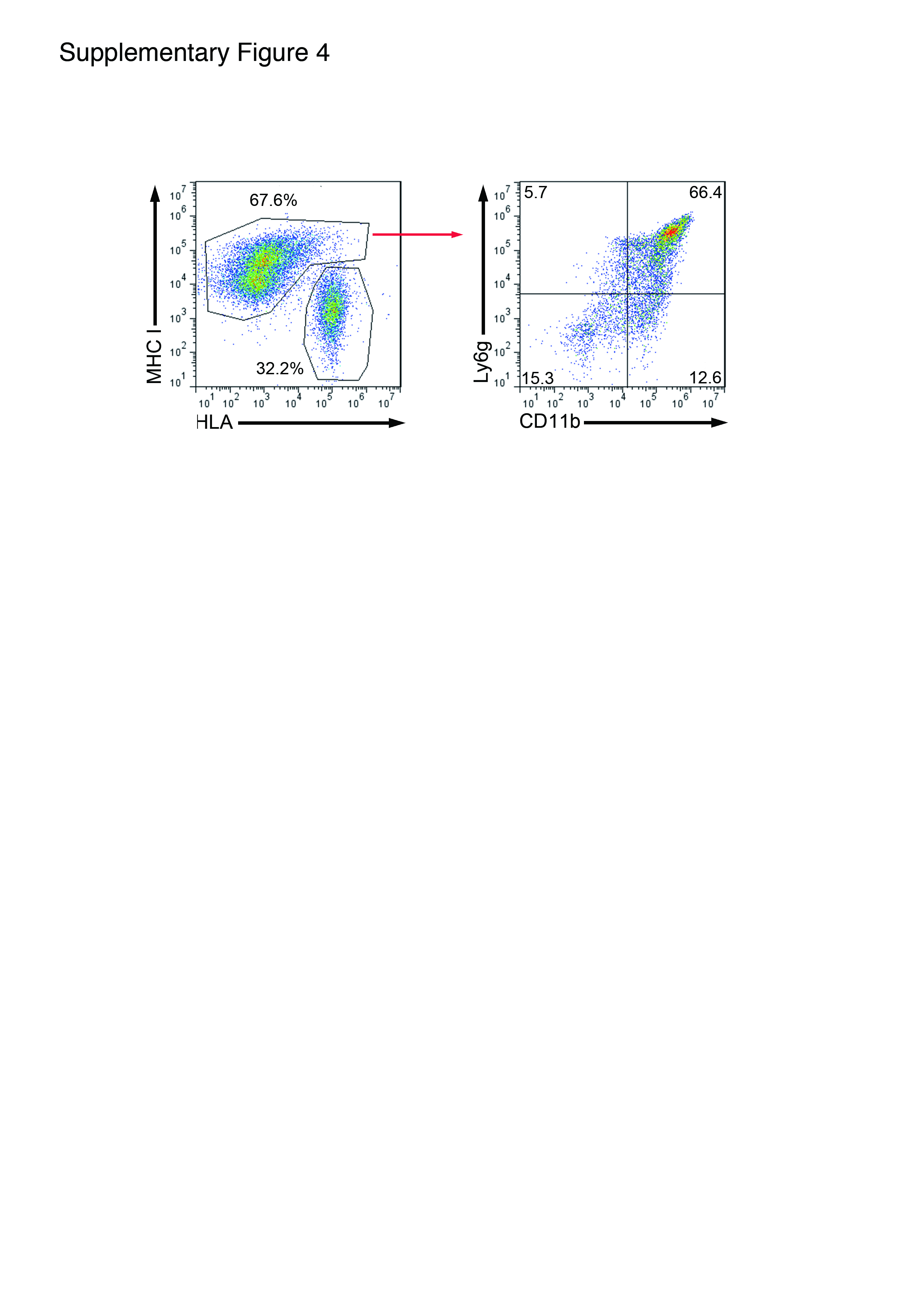

Supplement: Additional file 6: Figure S4. — Tumors dissected from NSCLC PDX mice contained the bone marrow-derived cells of the hosts. Representative plots of FACS analysis show dissociated cells from the tumors contained both human cells (HLA+) and murine cells (MHC I+) that were further subjected for analysis of murine Ly6g and CD11b expression. [file 13045_2015_156_MOESM6_ESM.jpg]
