# Supplementary material for: Quantitative evaluation of the immunodeficiency of a mouse strain by tumor engraftments
Source: J Hematol Oncol. 2015 May 29;8:59. doi: 10.1186/s13045-015-0156-y (PMC4478639; doi:10.1186/s13045-015-0156-y)
Supplement: Additional file 3: Table S1. — The final TEI scores of NSI, NOD-scid, scid, nude, Rag2−/−, IL2Rg−/−, and WT mice measured by xenograft experiments. [file 13045_2015_156_MOESM3_ESM.docx]

**Supplementary Table 1. The final TEI scores of NSI, NOD-*scid*, *scid*, nude, *Rag2-/-, IL2Rg-/-*, and WT mice measured by xenograft experiments.**

| **Strain(s)** | **Cell line** | **TEI*_mouse_*** | | | **TEI*_xenograft_*** |
| --- | --- | --- | --- | --- | --- |
|  |  | **H** | **M** | **L** |  |
| **NSI** | K562 | 0.023 | 0.012 | 0.007 | 0.027 |
|  | A549 | 0.059 | 0.051 | 0.012 |  |
|  | xenograft | 0.041 | 0.031 | 0.010 |  |
| **NOD-*scid*** | K562 | 0.012 | 0.003 | 0.000 | 0.014 |
|  | A549 | 0.050 | 0.020 | 0.000 |  |
|  | xenograft | 0.031 | 0.011 | 0.000 |  |
| ***scid*** | K562 | 0.003 | 0.000 | 0.000 | 0.006 |
|  | A549 | 0.021 | 0.012 | 0.000 |  |
|  | xenograft | 0.012 | 0.006 | 0.000 |  |
| **nude** | K562 | 0.000 | 0.000 | 0.000 | 0.004 |
|  | A549 | 0.018 | 0.004 | 0.000 |  |
|  | xenograft | 0.009 | 0.002 | 0.000 |  |
| ***Rag2-/-*** | K562 | 0.000 | 0.000 | 0.000 | 0.002 |
|  | A549 | 0.015 | 0.000 | 0.000 |  |
|  | xenograft | 0.007 | 0.000 | 0.000 |  |
| ***IL2Rg-/-*** | K562 | 0.001 | 0.000 | 0.000 | 0.001 |
|  | A549 | 0.006 | 0.000 | 0.000 |  |
|  | xenograft | 0.003 | 0.000 | 0.000 |  |
| **WT** | K562 | 0.000 | 0.000 | 0.000 | 0.000 |
|  | A549 | 0.000 | 0.000 | 0.000 |  |
|  | xenograft | 0.000 | 0.000 | 0.000 |  |

**Supplementary Table 1. The final TEI scores of NSI, NOD-*scid*, *scid,* nude, *Rag2-/-, IL2Rg-/-*, and WT mice measured by xenograft experiments.** The TEI scores of each individual NSI, NOD-*scid*, *scid*, nude, *Rag2-/-*, *IL2Rg-/-*, and WT mice that were injected with 1×10^6^ (high), 1×10^5^ (medium), and 1×10^4^ (low) K562-GFP (TEI_K562_) or A549 cells (TEI_A549_). The final TEI scores (TEI_xenograft_) were the average of TEI_K562_ and TEI_A549_. The data represent the means +/- s.e.m.
