# Supplementary material for: Quantitative evaluation of the immunodeficiency of a mouse strain by tumor engraftments
Source: J Hematol Oncol. 2015 May 29;8:59. doi: 10.1186/s13045-015-0156-y (PMC4478639; doi:10.1186/s13045-015-0156-y)
Supplement: Additional file 4: Table S2. — The final TEI score of NSI, NOD-scid, IL2Rg−/−, Rag2−/−, scid, nude and WT mice measured by allograft experiments. [file 13045_2015_156_MOESM4_ESM.docx]

**Supplementary Table 2.  The final TEI score of NSI, NOD-*scid, IL2Rg-/-*, *Rag2-/-*, *scid*, nude and WT mice measured by allograft experiments.**

| **Strain(s)** | **Cell line** | **TEI*_mouse_*** | | | **TEI*_allograft_*** |
| --- | --- | --- | --- | --- | --- |
|  |  | **H** | **M** | **L** |  |
| **NSI** | RMA | 0.154 | 0.096 | 0.060 | 0.070 |
|  | B16F10 | 0.068 | 0.032 | 0.009 |  |
|  | allograft | 0.111 | 0.064 | 0.035 |  |
| ***IL2Rg-/-*** | RMA | 0.065 | 0.038 | 0.034 | 0.029 |
|  | B16F10 | 0.031 | 0.009 | 0.000 |  |
|  | allograft | 0.048 | 0.023 | 0.017 |  |
| **NOD-*scid*** | RMA | 0.044 | 0.031 | 0.008 | 0.027 |
|  | B16F10 | 0.061 | 0.018 | 0.000 |  |
|  | allograft | 0.053 | 0.024 | 0.004 |  |
| ***scid*** | RMA | 0.015 | 0.006 | 0.000 | 0.013 |
|  | B16F10 | 0.051 | 0.014 | 0.000 |  |
|  | allograft | 0.031 | 0.008 | 0.000 |  |
| ***Rag2-/-*** | RMA | 0.011 | 0.002 | 0.000 | 0.013 |
|  | B16F10 | 0.048 | 0.010 | 0.000 |  |
|  | allograft | 0.031 | 0.008 | 0.000 |  |
| **nude** | RMA | 0.005 | 0.000 | 0.000 | 0.008 |
|  | B16F10 | 0.026 | 0.007 | 0.000 |  |
|  | allograft | 0.031 | 0.007 | 0.000 |  |
| **WT** | RMA | 0.000 | 0.000 | 0.000 | 0.002 |
|  | B16F10 | 0.015 | 0.002 | 0.000 |  |
|  | allograft | 0.008 | 0.001 | 0.000 |  |

**Supplementary Table 2. The final TEI scores of NSI, NOD-*scid*, *scid,* nude, *Rag2-/-, IL2Rg-/-*, and WT mice measured by allograft experiments.** The TEI scores of each individual NSI, *IL2Rg-/-*, NOD-*scid*, *scid*, *Rag2-/-*, nude, and WT mice that were injected with 1×10^6^ (high), 1×10^5^ (medium), and 1×10^4^ (low) K562-GFP (TEI_RMA_) or A549 cells (TEI_B16F10_). The final TEI scores (TEI_allograft_) were the average of TEI_RMA_ and TEI_B16F10_. The data represent the means +/- s.e.m.
